# Investigating the Role of Large-Scale Domain Dynamics in Protein-Protein Interactions

**DOI:** 10.3389/fmolb.2016.00054

**Published:** 2016-09-13

**Authors:** Elise Delaforge, Sigrid Milles, Jie-rong Huang, Denis Bouvier, Malene Ringkjøbing Jensen, Michael Sattler, Darren J. Hart, Martin Blackledge

**Affiliations:** ^1^Institut de Biologie Structurale, CEA, Centre National de la Recherche Scientifique, University Grenoble AlpesGrenoble, France; ^2^Institute of Structural Biology, Helmholtz Zentrum MünchenNeuherberg, Germany; ^3^Center for Integrated Protein Science Munich at Biomolecular NMR, Technische Universität MünchenGarching, Germany

**Keywords:** multi-domain proteins, free-energy landscape, conformational dynamics, nuclear magnetic resonance (NMR), small angle scattering, chemical exchange saturation transfer (CEST), single molecule Förster resonance energy transfer (FRET)

## Abstract

Intrinsically disordered linkers provide multi-domain proteins with degrees of conformational freedom that are often essential for function. These highly dynamic assemblies represent a significant fraction of all proteomes, and deciphering the physical basis of their interactions represents a considerable challenge. Here we describe the difficulties associated with mapping the large-scale domain dynamics and describe two recent examples where solution state methods, in particular NMR spectroscopy, are used to investigate conformational exchange on very different timescales.

Over the last four decades, X-ray crystallography, NMR and increasingly electron microscopy have provided unique insight into the nature of functionally-essential interactions between a vast array of biologically active molecules. This remarkable success has often overlooked the importance of the molecular motions that are required for function, with structural biologists tending to focus their attention on highly stable, high affinity biomolecular interactions. Proteins are however intrinsically dynamic, they can exhibit conformational modes of vastly differing amplitudes, from local bond fluctuations to folding/unfolding transitions, on timescales varying from femtoseconds to days. Interactions between proteins are also often required to be weak, for example in signaling pathways where efficiency and reversibility of information transfer are of key importance. The resulting focus on interactions that are strong enough to allow structure determination in terms of a single set of three-dimensional coordinates thus provides a distorted perspective on the interactome.

In order to fully understand the molecular basis of interactions between physiological partners it is necessary to map the modulation of the free energy landscapes of the interacting molecules throughout the interaction trajectory. This aim is the focus of considerable interest, requiring experimental, theoretical and analytical development. For this reason solution state methods have been increasingly used to actively investigate the nature of transient interactions between biomolecules (Vaynberg and Qin, 2006; Sugase et al., 2007; Bashir et al., 2010; Salmon et al., 2011).

Such considerations are particularly relevant for a highly abundant class of proteins whose physical characteristics are defined by their dynamic nature. The unexpected discovery that a high fraction of eukaryotic, but also prokaryotic and viral genomes code for proteins domains whose functional state is natively unfolded (Dyson and Wright, 2005; Uversky and Dunker, 2010), has imposed a new perspective on our understanding of molecular recognition. In contrast to folded proteins, the primary sequence of intrinsically disordered proteins (IDPs) does not code for a single, energetically stable fold, but occupies a flatter free-energy surface, spanning a continuum of different conformations. The dynamic nature of IDPs allows access to functional modes that are otherwise inaccessible to folded proteins. For example, the dynamic intrinsically disordered domains that fill the nuclear pore (nucleoporins), exhibiting fast association and dissociation rates with nuclear transport receptors, thereby facilitating highly specific but rapid transport of cargoes into the nucleus via a continuum of ultra-weak interactions (Milles et al., 2015). Interactions between IDPs often show kinetics that are more complex than simple two-state mechanisms, remaining dynamic within the complex (Kragelj et al., 2015), exhibiting “fuzzy” interactions that involve for example local conformational funneling into partner-specific bound conformations (Schneider et al., 2015), or involving transient, non-specific interactions to enhance affinity (Fink, 2005; Dunker et al., 2008; Tompa and Fuxreiter, 2008; Wright and Dyson, 2009; Van Roey et al., 2012; Forman-Kay and Mittag, 2013; Kosol et al., 2013).

The specific subject of this mini-review concerns the large family of proteins that comprise both folded and intrinsically disordered domains. Multi-domain proteins are ubiquitous in all studied interactomes (Vogel et al., 2004). They are characterized by large-amplitude motions that play important roles in almost every aspect of biomolecular function (Bahar et al., 2007; Smock and Gierasch, 2009; Tzeng and Kalodimos, 2011; Mackereth and Sattler, 2012). Such large-scale modes are often mediated by intrinsically disordered linkers that define the conformational freedom available to the different domains (Shamoo et al., 1995; Ma et al., 2011). Solution-state approaches are essential to probe the ensemble of conformational states sampled by such complex macromolecules. In particular, nuclear magnetic resonance (NMR), small-angle X-ray scattering (SAXS) and single molecule Förster resonance energy transfer (FRET) can be used to investigate the nature of the ensembles of interchanging conformers present in solution (Baber et al., 2001; Margittai et al., 2003; Bernadó et al., 2004, 2007; Henzler-Wildman et al., 2007; Ryabov and Fushman, 2007; Boehr et al., 2009; Clore and Iwahara, 2009; Bashir et al., 2010; Bernadó and Blackledge, 2010; Bertini et al., 2010; Rambo and Tainer, 2010; Boura et al., 2011; Mackereth et al., 2011; Rózycki et al., 2011; Camilloni et al., 2012; Mackereth and Sattler, 2012; Rezaei-Ghaleh et al., 2013; Russo et al., 2013; Hennig et al., 2014).

The highly dynamic nature of multi-domain proteins necessitates the development of analytical approaches for the interpretation of the available experimental data in terms of representative ensembles. Although the description of the available conformational space already represents a demanding task, due to the risk of over-fitting, a second, equally formidable requirement is the estimation of the populations of different sub-states and, if possible, their rates of interconversion. The time-scales of this dynamic exchange, and the conformational dynamics that occur within different sub-states also define the details of the analytical approaches that can be applied, rendering the task yet more daunting.

Here, we will discuss the application of NMR, SAXS, and single molecule FRET to the study of the conformational sampling of two highly flexible multi-domain proteins, the human U2AF65 protein that plays an essential role in the spliceosome assembly (Banerjee et al., 2003; Wahl et al., 2009; Mackereth et al., 2011), and the 627-NLS domain of the PB2 segment of influenza polymerase (Tarendeau et al., 2008; Delaforge et al., 2015). These two cases illustrate very different exchange regimes, impacting the appropriate interpretation of the experimental data.

NMR spin relaxation is potentially a very powerful means to describe both local and global dynamics of macromolecules in solution. Nevertheless, quantitative analysis of the conformational space sampled by two domains relative to each other is highly challenging (Baber et al., 2001; Wong et al., 2009; Ryabov et al., 2012; Xia et al., 2013). More often, paramagnetic relaxation enhancements (PREs) are used to detect weakly populated close contacts between domains, for example transient encounter contacts in protein-protein and protein-nucleic acid complexes (Iwahara et al., 2004; Tang et al., 2006; Volkov et al., 2006). The level of information can be enhanced considerably if the paramagnetic center exhibits anisotropic magnetic susceptibility, in which case pseudo-contact shifts are induced that are sensitive to the distribution of distances and orientations of vectors connecting the observed spins and the electron spin, relative to the susceptibility tensor. Under such conditions, or when dissolved in a dilute liquid crystalline solution, residual dipolar couplings (RDCs) can also be measured to provide information about the distribution of orientations of the structured domains relative to each other (Tolman and Ruan, 2006; Salmon and Blackledge, 2015). In addition, SAXS reports on the pairwise distribution functions of all conformations averaged over the ensemble (Bernadó et al., 2007).

Phenomenologically, large levels of conformational disorder are often manifest by the inability to interpret the experimental data in terms of a single conformation, so that it becomes necessary to invoke the presence of multiple conformations present simultaneously in solution. Substantial efforts targeting ensemble descriptions of flexible multi-domain proteins have been directed toward using NMR and SAXS data to account for this conformational heterogeneity. As mentioned above, care must be exercised in order to avoid over-fitting and to introduce some estimate of uncertainty of populations and representative conformers in the ensemble descriptions. Examples of recent analytical approaches include an estimation of the maximum occurrence of each possible protein conformation on the basis of experimental NMR or SAXS data (Bertini et al., 2007, 2010; Ravera et al., 2014), or weighted-ensemble selection from molecular dynamics (MD) simulation or available crystal structures (Yang et al., 2010; Francis et al., 2011; Russo et al., 2013). Conformational space can also be sampled using replica exchange MD (Sgourakis et al., 2007; Wu et al., 2009; Terakawa and Takada, 2011; Knott and Best, 2012; Narayanan et al., 2012; Zhang et al., 2012; Mittal et al., 2013; Wang et al., 2013) using the experimental data as constraints to guide ensemble distributions that reproduce the experimental data on average (Im et al., 2012; Cavalli et al., 2013; Roux and Weare, 2013). Alternatively rigid-body modeling can be used to develop representative ensembles of conformers (Deshmukh et al., 2013).

Conformational sampling can be achieved with high efficiency using restraint-free Monte-Carlo approaches, exploiting for example statistical coil models to describe the backbone dihedral angle distributions of the inter-domain linker. The nature of the intrinsic conformational equilibrium can then be examined by generating representative ensembles of conformers, using an adaptation of the ASTEROIDS approach for ensemble representations of intrinsically disordered systems (Nodet et al., 2009; Salmon et al., 2010; Ozenne et al., 2012; Guerry et al., 2013). Comparison with the experimental data is then used to identify sub-ensembles of conformers that, in combination, represent the Boltzmann distribution in solution. Ensemble descriptions of highly disordered systems are faced with two problems; identification of representative conformational states, and determination of their relative populations. ASTEROIDS uses a genetic algorithm to identify combinations of conformational states that when considered together reproduce the experimental data within estimated experimental uncertainty. Different conformers (*i*) have populations given by *p*_*i*_ = 1/*n* where *n* is the number of conformers in the ensemble. Populations are not optimized, so that if a given state requires a higher weight to fulfill experimental data, additional conformers with similar characteristics will be present. The optimal number of structures necessary to reproduce the complexity of the experimental data can be estimated by cross-validation of independent experimental data that are not used in the analysis (Salmon et al., 2010; Guerry et al., 2013; Huang et al., 2014). The single-step selection of ASTEROIDS avoids additional optimization of the weights of specific conformers, and is therefore compatible with robust statistical analysis allowing estimation of the confidence levels of both conformation and population.

This approach was adapted to map the free-energy landscape of the RNA Recognition Motifs RRM1 and RRM2 domains of the U2AF65 protein. The two RRMs are connected by a flexible linker and adopt multiple domain arrangements as indicated by a combined analysis of PRE, RDC and SAXS data (Huang et al., 2014). The analysis reveals a heterogeneous ensemble of states dominated by highly populated, but very different relative positions of RRM1 and RRM2, despite the fact that these conformations are not strongly represented in the unrestrained ensemble of states. These conformations were found to resemble previously proposed “closed” and “open” states (Madl et al., 2010; Simon et al., 2010; Mackereth et al., 2011; Hennig et al., 2015), where the latter corresponds to the RNA-bound form of the protein (Figure 1), supporting the role of conformational selection from the free-state ensemble as a driving force of this interaction (Mackereth et al., 2011; Mackereth and Sattler, 2012). “Open” and “closed” conformations were found to lie within a continuous density of states, indicating that transition could occur between these states without invoking large conformational jumps. Inspection of the nature of the interacting surfaces dominating the ensemble suggested that the “closed” state was stabilized by electrostatic interactions. This prediction was supported by an observed weakening of transient contacts in this interface, as detected by a reduction of distinctive PREs with increasing ionic concentration, providing an independent support for the nature of the solution state ensemble that is derived uniquely from experimental data.

**Figure 1:**
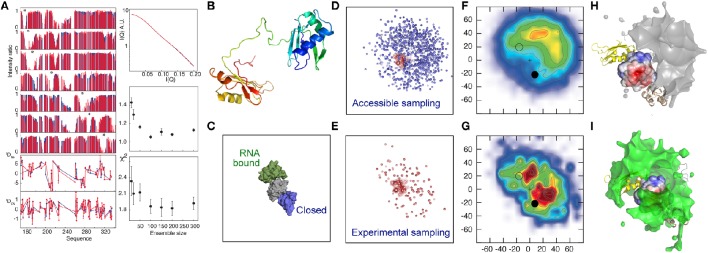
**Electrostatic interactions dominate the conformational equilibrium populated by the multi-domain splicing factor U2AF65. (A)** Comparison between fitted (blue) and experimental (red) data. Upper left panel—PRE intensity ratios, lower-left panel, RDCs, top right panel SAXS, middle right panel active χ^2^, lower right panel cross validated χ^2^, both with respect to ensemble size. **(B)** Cartoon description of RRM1 and RRM2 domains, connected by the 32 amino-acid flexible linker. **(C)** Position of the previously determined closed (2YH0-blue) and open (2YH1-green) conformations of RRM1 relative to RRM2 (Mackereth et al., 2011). **(D–I)** Representation of the accessible (prior) conformational sampling of RRM2 compared to RRM1 on the basis of statistical coil sampling of the linker **(D,F,H)** and on the basis of experimental data **(E,G,I)**. **(D,E)**—Centre-of-mass representation, **(F,G)**—2D projections onto XY and XZ planes (in Å) with populations (derived from 100 Monte Carlo simulations; χ^2^/N≈1, red/blue highly/weakly populated. “+” indicates center-of-mass of RRM1; closed and open circles 2YH0 and 2YH1, respectively, **(H,I)**—3D density maps (5% population contour showing the probability of the center of mass position averaged over the ensemble) of RRM2 distribution with respect to RRM1, showing occlusion of the acidic patch on the surface of RRM1 in the experimental distribution that is not present in the prior sampling, demonstrating that the experimental data require a redistribution of sampling. (Reprinted with permission from Huang et al., 2014. Copyright 2014 American Chemical Society).

In the latter example the experimental data are assumed to report on an ensemble of conformations that are in rapid exchange with respect to the difference in NMR chemical shifts, RDCs and PREs. In effect this assumes that all conformations exchange on timescales faster than or equal to tens of microseconds. Interpretation of the experimental data calculated for each independent sub-state can then be averaged to assess the ability of the ensemble to reproduce the experimental data. This exchange regime will not always be respected, for example in the case of strong interactions between domains, where high activation energies of dissociation may result in much slower exchange between states. Such an exchange regime was recently studied in the 627-NLS multi-domain component of Influenza A polymerase.

The viral RNA polymerase complex is made up of three separate proteins, PA (acidic protein), and PB1 and PB2 (basic proteins 1 and 2) that are imported into the host nucleus following translation to assemble into new polymerase heterotrimers that further catalyze viral RNA replication and transcription. PB2 enters the nucleus by binding to Importin α (Impα) via its C-terminal domain, termed the 221 amino-acid 627-NLS domain (Tarendeau et al., 2008; Kuzuhara et al., 2009) containing a nuclear localization signal (NLS) peptide. The 627-NLS domain is of particular interest, as a large proportion of mutations characterizing adaptation of avian viruses to human hosts are located on its surface (Tarendeau et al., 2008; Mehle and Doudna, 2009). Crystal structures of 627-NLS have been determined, in isolation from both avian and human forms of Influenza (Tarendeau et al., 2008), and more recently in the context of the entire polymerase complex (Pflug et al., 2014), in all cases exhibiting effectively identical compact conformations. This conformation was, however, incompatible with binding of the NLS domain to Impα, suggesting that large-scale conformational rearrangement of either 627-NLS or Impα would be necessary for successful interaction (Boivin and Hart, 2011).

A recent NMR study (Delaforge et al., 2015) of 627-NLS unambiguously revealed the presence of a more complex conformational equilibrium in solution, with approximately twice as many peaks present in the ^1^H-^15^N TROSY spectrum as expected, and one set of peaks exhibiting the same chemical shifts as the 627 and NLS domains in isolation. This spectrum is characteristic of slow exchange (in this case *k*_ex_ = 30 s^−1^ at 15°C) between a closed form of the protein (the crystalline conformation) and a previously uncharacterized open form where the two domains retain their secondary and tertiary structure, but dislocate, and evolve independently of each other (Figure 2). One set of peaks therefore report on the ensemble of conformations that are in fast exchange, while the other set of peaks report on the closed state that is stabilized by a tripartite salt bridge implicating highly conserved basic and acidic amino acids in the interface between the 627 and NLS domains, and in the 13-amino acid inter-domain linker. The linker becomes flexible upon dislocation, and, as in the case of the U2AF65 RRM1-RRM2 domains, provides the degrees of freedom required for the large-scale domain dynamics characterizing the open form.

**Figure 2:**
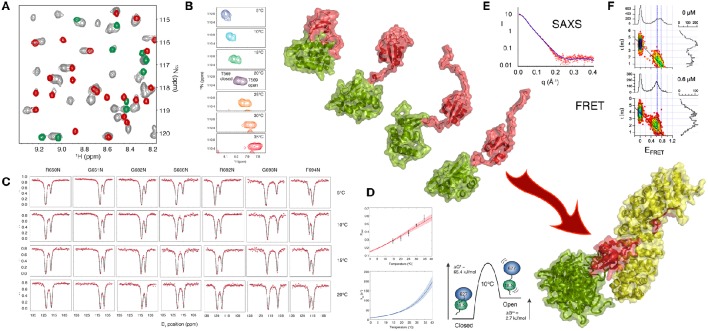
**Large Scale Conformational Dynamics Control Influenza Polymerase PB2 627-NLS domain Binding to Importin α. (A)** The first evidence that the 627-NLS domain of influenza polymerase PB2 subunit samples two conformations in solution is provided by the ^15^N-^1^H correlation spectrum that exhibits two sets of peaks in solution (gray), one set corresponds to resonance positions of either the free 627 (green) or the free NLS (red) domains, suggesting that the two-domain protein exchanges between open and closed forms in solution. **(B)** This exchange is temperature dependent, with increasing population of the open form at higher temperatures as shown in this example showing two peaks reporting on the environment sensed by T569 in the two (open and closed) forms of the protein. The majority of peaks show such behavior, with a large range of ^15^N and ^1^H shifts between the two forms. **(C)** Chemical exchange saturation transfer (CEST) of seven resolved peaks, showing “dips” reporting on the open and closed forms of the protein. **(D)** CEST profiles provide information about population, exchange rates and structure, and simultaneous analysis of all spectra in **(C)** allows for the determination of enthalpic and entropic contributions to the equilibrium thermodynamics. **(E)** SAXS of the complex suggests localization of the 627 domain in the vicinity of the C terminus of Importin α. **(F)** smFRET also shows that 627-NLS exhibits domain dynamics in the free form, and demonstrated that only the open form remains in the bound form equilibrium (concentration of titrated Importin is shown in the panels). Large-scale domain dynamics are therefore essential for binding to Importin α. (Reprinted with permission from Delaforge et al., 2015. Copyright 2015 American Chemical Society).

Single molecule FRET can also be used to probe the distance distribution and inter-domain dynamics of the two folded domains of 627-NLS. Two populations reporting on the open and closed forms are again observed. The estimated timescale of the different motional modes, characterized by fast interconversion within the open state (τ_ex_ < 50 μs) and slow interconversion between the open and the closed states (τ_ex_ > 20 ms) also confirms the similarity of the closed state to the crystal conformation, showing negligible rapid motion, and the presence of significant fast domain dynamics in the open state, in good agreement with the NMR analysis.

Exchange between open and closed conformations of 627-NLS was characterized using NMR lineshape analysis and Chemical Exchange Saturation Transfer (CEST), revealing a strong temperature dependence, ranging from 10 to 100 s^−1^ over the range 5–30°C, with the population of the open form increasing significantly from predominantly closed at 5°C (*p* = 0.2) to approximately equal populations at 30°C. Small angle X-ray and neutron scattering data were also measured over the entire temperature range, resulting in very good agreement between expected population-weighted scattering curves from open and closed ensembles and the experimentally determined populations from NMR exchange analysis. Simultaneous analysis of CEST data from multiple different sites throughout the protein as a function of temperature, using an Eyring relationship, provides unique insight into the thermodynamics of the temperature-dependent equilibrium. The activation energy of opening and closing is dominated by enthalpic contributions, while the open state exhibits both entropic and enthalpic contributions compared to the closed state, suggesting additional non-specific non-bonding interactions between the surfaces, as in the case of RRM1/RRM2. Characterization of the temperature-dependence of the equilibrium is of functional interest because viral replication involves adaptation from the warmer bird intestine to the cooler human respiratory system (Massin et al., 2001). Comparison of the temperature dependence of reconfiguration dynamics of 627-NLS from human and bird-adapted proteins is currently underway in our laboratories.

The physiological interaction between 627-NLS and Impα was also investigated using solution methods. The population of the closed form of the protein disappears upon interaction, as shown using single molecule FRET, with the open form maintaining the characteristics of fast distance fluctuations between the attached dyes and therefore suggesting fast inter-domain dynamics in the bound state. Although SAXS measurements of the complex also indicate flexibility of 627 with respect to Impα when bound to NLS, they also reveal that the 627 domain is localized primarily in the vicinity of the C terminus of Impα, possibly stabilized by interactions with the 34 amino acid long intrinsically disordered C terminal tail of Impα.

These observations strongly suggest that while the closed form is known to be necessary for function within the polymerase-RNA complex (Pflug et al., 2014), the open form of 627-NLS is required for interaction with Impα. Interestingly, mutation of R650A in the 627 domain removes the possibility of forming the salt bridge, resulting in suppression of the closed form in solution. This same mutation was independently shown to abrogate polymerase activity in the nucleus, while still allowing for nuclear import (Kirui et al., 2014), substantiating our model whereby the open-form mediates nuclear import. Further structural investigations of different forms of the RNA polymerase complex suggest that an open 627-NLS may also play additional roles in remodeling the polymerase structure during the viral cycle (Hengrung et al., 2015; Thierry et al., 2016).

The two studies described above demonstrate the functional importance of large-amplitude dynamics mediated by the intrinsically disordered linker peptides in multi-domain assemblies. In both cases the analysis suggests a role for conformational selection from an intrinsic pre-existing equilibrium in the interaction with physiological partners. Both studies show how solution-state approaches can be used to understand the role of complex dynamic conformational equilibria in biomolecular function and, more specifically, that these mechanisms could not be understood without a detailed description of the ensemble of states sampled in solution.

## Author contributions

All authors listed, have made substantial, direct and intellectual contribution to the work, and approved it for publication.

## Funding

This work was funded by the Agence Nationale de la Recherche (ANR) under ComplexDynamics (SIMI7 – MB). SM acknowledges funding from an EMBO long-term fellowship (ALTF 1234-2014) and EC (EMBOCOFUND2012, GA-2012-600394) via Marie Curie Action. This work used the platforms of the Grenoble Instruct Centre (ISBG; UMS 3518 CNRS-CEA-UJF-EMBL) with support from FRISBI (ANR-10-INSB-05-02) and GRAL (ANR-10-LABX-49-01) within the Grenoble Partnership for Structural Biology (PSB).

### Conflict of interest statement

The authors declare that the research was conducted in the absence of any commercial or financial relationships that could be construed as a potential conflict of interest.
